# A study of *Rashtriya Swasthya Bima Yojana* in Chhattisgarh, India

**DOI:** 10.1186/1753-6561-6-S1-O5

**Published:** 2012-01-16

**Authors:** Sulakshana Nandi, Kanica Kanungo, Md Hashim Khan, Haripriya Soibam, Tarang Mishra, Samir Garg

**Affiliations:** 1 Public Health Resource Network, India; 2 Student at Jamia Hamdard University, New Delhi, India

## Introduction

The *Rashtriya Swasthya Bima Yojana* (RSBY) is a national health insurance scheme launched in 2008 by government of India to provide health insurance to households living below poverty line (BPL) in order to protect them from major health shocks that involve hospitalisation. In Chhattisgarh RSBY was launched in June 2009 and Durg was the first district to be enrolled under this scheme.

In this paper, we attempt to analyse implementation of RSBY in Durg, Chhattisgarh and explore whether out-of-pocket expenses are still incurred by patients utilising this insurance.

## Methods

We selected Durg district as the study site as it has the highest utilisation rate in Chhattisgarh. We conducted the exit interviews of patients who used RSBY in months of May and June 2010 at RSBY empanelled hospitals. As per the RSBY utilisation data from the district, only two government hospitals (district hospital at Durg and community health centre at Gunderdehi) had undertaken hospitalisations. We included both theses hospitals. Of private hospitals, we selected hospitals in Durg and Bhilai towns through convenience sampling. Hence two public hospitals (out of 16 empanelled) and five private hospitals (out of 10 empanelled) were selected for the study.

We spent four days at each selected hospital and attempted exit interviews of 10 cases per hospital. However the sample per hospital varied due to non-cooperation by some of the hospitals. Furthermore, the RSBY facility was closed for most of the data collection period (June 2010 at community health centre, Gunderdehi). In order to complete the sample, we identified the villages with highest number of hospitalisations and traced RSBY beneficiaries through Mitanins (a village level female health worker).

We used a structured questionnaire to conduct exit interviews of patients at the selected healthcare facilities and of RSBY beneficiaries in the village. We pilot tested the questionnaire on five patients before using it for sample population. In total we interviewed 100 RSBY beneficiaries that represent 4% of total hospitalised cases in Durg district and 2% of total hospitalised cases in Chhattisgarh in that period. We collected secondary data from RSBY website.

## Results

In Chhattisgarh 46% of the eligible families were enrolled under RSBY till July 2010. Hospitalisation rate was only two per 1000 persons enrolled. The insurance claim ratio was low with INR 64000000 (USD 1374280) paid as claims so far, whereas insurance companies received an annual premium of INR 750000000 (USD16104800). The average value of hospitalisations in Chhattisgarh is INR 4411 (USD 94.7).

In our sample population, we found that only 4% of respondents had received their RSBY smart card on the spot. People were hardly given any information on RSBY. More people from rural areas and those belonging to scheduled castes and scheduled tribes were using government hospitals. *Mitanins* (a village level female health worker) refereed 40% of patients using government hospitals. Main symptoms that brought people to the health facility included weakness in 33% of cases; fever in 18% of cases; surgery in 13% of cases and, abdominal pain in 10% of cases.

For people who were aware of the amount deducted, the average value of hospitalisation was INR 4988 (USD 107.1) in government healthcare services and INR 7416 (USD 159.2) in private healthcare services. We found that 58% of the respondents who used private healthcare services and 17% of those who used government healthcare services incurred out-of-pocket expenses. Average out-of- pocket expenditure was INR 1078 (USD 23.1) in private sector and INR 309 (USD 6.6) in government sector. Most private hospitals fixed a quota for BPL patients, beyond which they refused to admit patients under RSBY.

## Discussion

RSBY is meant for the poorest and aims to relieve them of the burden of healthcare costs. However, our findings suggest that patients still incurred out-of-pocket expenses. Furthermore, most private hospitals did not admit patients under RSBY beyond their BPL quota.

A lack of transparency at healthcare facility level is evident as a large number of persons were not aware of the amount deducted from their RSBY cards. There is need to enhance transparency and proactive disclosure by healthcare facilities for patients and for effective analysis of the scheme.

There is need to strengthen government healthcare services and regulate private healthcare services in order to have desired results of RSBY.

